# Book Reviews

**Published:** 1799-09

**Authors:** 


### CRITICAL RETROSPECT

OF

### *MEDICAL AND PHYSICAL LITERATURE.*

[FOREIGN AND DOMESTIC.]

*** *Our desire of doing justice to medical works, without exception, and giving at least a concise account of every book in the various branches of Medicine, as soon as it is published, is often frustrated, because we are not acquainted with the time and place of its publication. We shall therefore esteem it a favour, if authors or publishers will communicate to us this information as early as possible: but we cannot, consistently with our plan, take notice of books which have not been published within the last twelve months.*

### NATURAL HISTORY.

*The Natural History of the Tea-tree, with Observations on the medical qualities of Tea, and on the Effects of Tea-drinking; with coloured Plates.* A new edition. By J. C. LETTSOM, *M. D.* 4to. 102 pp. Price 10s. 6d. *London*; Dilly.

The subject of this splendid and elaborate work, being now in general use among the inhabitants of this kingdom, as well as in many other parts of Europe, and constituting a large portion of our commerce, it cannot but afford pleasure to the curious and intelligent, to possess the history of a shrub, with the leaves of which they are so well acquainted.

---

*Dissertationes academicæ Upsaliæ habitæ sub præsidio Caroli Petri Thunberg,* Equit. Reg. ord. Wasaei, &c. *Volumen primum.* Cum. Tab. V. aencis. 8vo. 326 pp. 1799. *Gœttingen*; Dieterich.

Whoever is acquainted with the name of THUNBERG, and the difficulty of procuring foreign dissertations, will no doubt consider himself indebted to M. PERSOON, whose name we find subjoined to the preface of this volume, as the collector and editor. Every friend of medical botany must feel a lively interest in the continuation of this valuable collection, which on account of its variety, and the useful species of information it conveys, cannot fail to meet with universal approbation. But we think it our duty to remind the respectable publisher of this work, not to neglect the promised plates, or even to forget them, as is the case in the volume before us, which contains the following dissertations: *Genera nova plantarum,* p. 1—8, 1781—1798. *De scientia botanica utili atque jucunda,* 1793. *De Flora strengnesensi,* 1791. *De use Menyanthidis trifoliatæ,* 1797. *De oleo Cajeputi,* I. II. 1797. *De Moxæ atque Ignis in Medicina usu,* 1788, *De cortice Angusturæ,* 1793. *De arbore Toxicaria Macassariensi,* 1788. *De Medicina Africanorum,* 1785. *Observationes circa remedia nonnulla indigena.* 1790. *De nautarum valetudine tuenda,* 1795. *Observationes in Pharmacopœam suecicam,* I. 1796.

*Herbarium*

*Herbarium vivum Muscorum frondosorum cum descriptionibus analyticis ad normam Hedwigii*, Pars I. Curante ALBERTO HOSE. 8vo. 93 pp. (Price two rix-dollars and nine grosch. Sax. Curr.) 1799. *Leipzig;* Græff.

Our expectations respecting the execution of this work have been rather disappointed. From the plausible title, we expected to find some new and peculiar arrangement and description of the mosses, so that the characters given to them by HEDWIG, might be more obvious and easy to the naturalist than he has hitherto been accustomed to meet with. But here, the mosses are merely pasted to coloured octavo pages, violet, blue, or red; without giving a specific description of their parts of generation; especially of those whose exterior difference and form are discoverable by the naked eye: nor has the author furnished us with a description of the distinguishing parts of such mosses as cannot be easily determined, without knowing their peculiar brim, cover, capsule, &c. All these requisites being neglected, Mr. HOSE has prefixed an index of his genera, a few synonymes, and an account of their native places, together with the time of their fructification. The number before us contains the following twelve species: *Tab.* 1. *Bryum argenteum.* 2. *Dicranum scoparium.* 3. *Fissidens pulvinata.* 4. *Hedwigia pulvinata.* 5. *Hypnum velutinum.* 6. *Hypnum intricatum.* 7. *Hypnum purum.* 8. *Leskea subtilis.* 9. *Polytrichum urnigerum.* 10. *Tetraphis pellucida.* 11. *Tortula muralis.* 12. *Trichostomum canescens.*

---

*Mineralogisches Taschenbuch*, &c. i. e. A Mineralogical Pocket-book, for the use of beginners and amateurs. By J. G. LENZ, Doctor of Philosophy and Professor at Jena. Vol. I. pages xxiv. and 300, besides Index and Tables, 1798. Vol. II. pages 246, in Twelves, 1799. *Erfurt;* Hennig.

The author's aim to guide the student and beginner, who have no opportunities of attending public lectures in this difficult branch of science, is indeed praiseworthy; but unfortunately there is something more than good-will required to succeed in any scientific attempt, without possessing either a peculiar talent for description, or genius for discovery. In justice, however, to the industrious author, it must be confessed that he has furnished an useful compend for the tyro; and though he cannot claim much merit of originality in the work itself, as he has chiefly adopted Werner's description of the external characters of fossils, yet his arrangement is sufficiently perspicuous and easy, especially to those pupils in mineralogy who have already acquired some elementary knowledge of this fascinating science.

In the first volume, Dr. Lenz treats of the different species of earths and stones; in the second, of the metals; and in the third he proposes to give an analytical account of the salts and inflammable substances.

---

*Journal für die Botanick*, i. e. The Botanical Journal: edited by SCHRADER, Counsellor of Health. Number first. 8vo. 272 pp. with three plates, 1799. *Göttingen;* Dieterich.

When the editor of a new Journal assures us in the introduction, that no attempt, or at least no successful one, has hitherto been made, which could answer the purpose of improving and extending this department of physical knowledge, and that he is enabled to remedy this deficiency, we ought naturally to expect that he will be thoroughly acquainted with the nature of his undertaking, so as to fulfil the promises by which he has committed himself to the public. He in a manner challenges competition, and invokes the severest shafts of the critic.

Our limits do not admit of analytical proof that M. Schrader is not strictly possessed of the qualifications requisite to a good botanical editor; for the very first of the essays entitled, *Lychenum gelantinesorum illustratio,* by Dr. Bernhardi, is not free from error and inaccuracy. Thus, instead of distinguishing particular species of the Lichen with care and attention, the author gives the *Lichen subtilis* a separate place, although it obviously is only the young Lichen *tenuissimus*, Diks. Fasc. I. T. II. Fig. 8.—farther, among the Lich. pulposos, he has confounded five different species; namely, the *Collema glaucescens, crispum, obscurum, cristatum et lobatum,* Hoffm.—as, on the other hand, the Lich. nigrescens he confounds with the *Collema auriculatum.*

The second article is written in German, by Dr. Nöhden, on the manner in which plants separate the pollen, or farina, from the parts of fructification;—the third, on the genus *usnea,* together with some remarks on Hoffman's Flora Germanica, by the editor, is likewise a very superficial performance.

The second section of this Journal consists of extracts from foreign botanical works, particularly Vahl's *Eclogæ Americanæ,* fol. 1726; Smith's botanical history of the *Mentha exigua*; Woodward's Remarks on the genus *Ulva,* and the Linnean Transactions, vol. iii. 1797.—The third section contains a review of several botanical publications; the fourth, literary correspondence; and the last, miscellaneous accounts. On the whole, it cannot be denied that this periodical work might prove equally interesting and useful to the friends of botanical science, if it were conducted with more ability and discernment.

